# Work, Stress, and Diurnal Bruxism: A Pilot Study among Information Technology Professionals in Bangalore City, India

**DOI:** 10.1155/2011/650489

**Published:** 2011-12-10

**Authors:** S. K. Rao, M. Bhat, J. David

**Affiliations:** ^1^Australian Research Centre for Population Oral Health (ARCPOH), School of Dentistry, University of Adelaide, Adelaide SA 5005, Australia; ^2^Department of Health Services Research and School of Dentistry, University of Liverpool, Waterhouse Building, 1-5 Brownlow Street, Liverpool L69 3GL, UK

## Abstract

The study assessed the prevalence of diurnal bruxism among information technology (IT) professionals and explored plausible predictors associated with the parafunctional habit. A cross-sectional study was designed and IT professionals were invited to participate. The inclusion criteria composed of participants in service for at least one year, having natural dentition, no history of cervical or facial injury and not undergoing orthodontic therapy. The participants (N = 147) were interviewed by a trained interviewer to record information. A pre-tested questionnaire that included questions related to work, stress symptoms and diurnal bruxism was completed by each participant. The prevalence of self-reported diurnal bruxism was 59%. Bivariate analyses revealed that work (*P* < 0.05) and work experience (*P* < 0.05) were significantly associated with self-reported diurnal bruxism. In the binary logistic regression analysis stress (Odds Ratio [OR]  = 5.9, 95% Confidence Interval [CI] 2.6–13.3) was identified to be a strong predictor of diurnal bruxism. Professionals with 11 or more years of experience were less likely to report diurnal bruxism (OR = 0.04, 95% CI 0.00–0.43) than those with 1 to 5 years of work experience. The study revealed that stress and less work experience were associated with diurnal bruxism among IT professionals in Bangalore city.

## 1. Introduction

Masticatory muscle activities can be normal or abnormal in nature and are categorized as *functional* (e.g., chewing, swallowing, and speaking) or *parafunctional* (e.g., tooth clenching, grinding, and various oral habits) [1]. Bruxism is one such parafunctional activity (PA) commonly seen in humans. It is defined as a diurnal or nocturnal PA that includes clenching, bracing, gnashing, and grinding of teeth [2, 3]. Awake or diurnal bruxism occurs during day time mainly as clenching [4], whereas nocturnal bruxism is a stereotyped movement of clenching and grinding type activity [3] and is mainly sleep related. 

Dental professionals in general are more concerned about bruxism due to its after effects on the oral and perioral structures. Bruxism occurs in healthy individuals and patients alike. Earlier studies indicate a wide variation in the prevalence of bruxism. According to a review by Seligman et al. [5] prevalence of bruxism and clenching in the general adult population ranged from 7% to 58% depending upon the type of investigation. Carvalho et al. [6] from their research reported that 50% of the police officers were bruxers either during sleep or awake. According to an investigation by Ahlberg et al. [7] about 11% of media personnel reported to have bruxism. Kuttila et al. [8] reported the occurrence of diurnal bruxism without symptoms in a population-based study to be 7.3%. 

Multiple factors have been identified to cause bruxism. Among them stress is increasingly considered as an initiating, predisposing, and perpetuating factor for bruxism, although their explicit relationship remains unclear. Several researchers concur that bruxism while awake or grinding during sleep is associated with stress and anxiety caused by family responsibilities or work pressure. A Brazilian study on police officers showed that bruxism was associated with emotional stress, independent of type of work [6]. A study from Finland delineated that an unequivocal linear association existed between self-reported bruxism and experience of severe stress exposure among multiprofessional media personnel. The positive association between continual stress and frequent bruxism was highly significant with an odds ratio of five [1]. Manfredini et al. [9] found an association with anxiety, depressive, and maniac symptoms in bruxers. 

Perhaps etiology of diurnal bruxism is considered to differ from nocturnal bruxism which occurs in relationship to arousal state. Van Selms et al. [10] suggested that the stress experienced was associated more with diurnal bruxism than nocturnal or sleep bruxism. According to Glaros [11] psycho-reactive stress could be a factor associated with day time clenching. Clenching of teeth has been reported during intense periods of increased workload [12]. Majority of the studies published in the literature are related to nocturnal or sleep bruxism compared to those related to diurnal or awake bruxism. Thus in this study diurnal bruxism was considered since this habit could be related to stress at work.

A unique subset of population is that of information technology (IT) professionals and presently they are an important workforce both in developed [13, 14] and developing countries. Since the 1990s there has been a steady increase in demand for IT in India and today she ranks as one of the largest outsourcer for occidental countries. There are approximately 916 IT providers registered with National Association of Software and Service Companies (NASSCOM) all over India [15]. A large number of companies are currently located in Bangalore city which is popularly known as the silicon valley of India. 

Jobs of IT professionals are highly skillful and to meet the challenges and deadlines, they usually work for extended hours and under such conditions “stress” is an inevitable consequence. Suparna et al. [16] from their investigation in India found that 35% of IT professionals were stressed and 78% experienced musculoskeletal problems. Despite this, studies exploring the stress experienced at work and its implications on oral health are lacking. Hence, we conducted this pilot study with the objective to assess the prevalence of diurnal bruxism among information technology professionals and to ascertain specific predictors associated with the parafunctional habit.

## 2. Materials and Methods

A cross-sectional study was conducted. Ethical clearance was obtained from the institutional review board of K.L.E. Institute of Dental Sciences and Hospital, Bangalore. The Human Relation's section of an IT industry was contacted first so as to obtain the company's permission. In total 256 IT professional worked in the company and were invited to participate in the study. Informed consent was obtained from those willing to participate in the study. The personal information collected during the study was kept confidential. The study period was from May 2007 to July 2007. Participants who had work experience in IT job for at least one year, having natural dentition, no history of cervical or facial injury, and not undergoing orthodontic therapy were included in the study. A trained person interviewed the participants to collect information. Specially designed pre-tested questionnaire was used to record data regarding work pattern, stress, and diurnal bruxism. The questionnaire was pilot-tested on 20 professionals none of whom were included in the main study. The questionnaire was in English since the participants were educated and could speak English fluently. It consisted of four parts; personal data, work inventory, stress symptom inventory [17], and diurnal bruxism. Work and stress categories were recorded on a five-unit scale (0 = never, 1 = a few times, 2 = regularly, 3 = frequently and 4 = always). Work inventory included questions on working for longer hours and tension during work. Stress symptoms inventory included symptoms like depression, strong heart beat, dry mouth, angry burst, inability to concentrate, weakness, fatigability, muscle stiffness in the morning, lock jaws, tight jaws, pain in the jaws, insomnia, headache, and excessive sweating. Parafunction—diurnal bruxism that included grinding and/or clenching of teeth during daytime—was recorded as either present (scored “1”) or absent (scored “0”). Personal data that were collected included their age, gender, and years of experience in IT industry.

## 3. Data Analysis

Statistical software SPSS 17.0 was used to analyze the data. Chi-square tests were carried out to find bivariate associations with age, gender, work experience, work, and stress with diurnal bruxism. Logistic regression analyses were performed to find if associations existed between age, gender, work experience, work, and stress with diurnal bruxism. Dependent and independent variables except work experience were dichotomized based on median split values at 50th percentile. The dichotomized variables were recoded by giving values 0 and 1. Work experience was classified into three categories: 1 to 5 years, 6 to 10 years, and 11 years or more. Recoding was done for the variable by assigning values 0, 1, 2 in the increasing order. The variables were then entered into a single model as categorical values. The probability with 95% confidence intervals was computed for these results. Cronbach's alpha was calculated for the reliability of different sets of questions in the questionnaire. A *P* value of less than 0.05 was considered to be statistically significant.

## 4. Results

A total of 147 professionals participated in the study (response rate = 57.0%). Out of 164 who volunteered to participate in the study 17 were excluded since they had work experience less than 1 yr (*n* = 9), partial denture (*n* = 5), undergoing orthodontic therapy (*n* = 2), and history of injury (*n* = 1). The characteristics of participants are shown in Table 1. Among them 72 (49.0%) were males. Age of the participants ranged between 20 and 60 years with a mean age of 33.90 ± 7.24 years and 28.84 ± 5.22 years for males and females, respectively. Participants were more in the age group of 30 to 39 years among males (59.7%) and in the age group of 20 to 29 years among females (69.3%). The Cronbach's alpha for the various items used in the questionnaire ranged between 0.66 and 0.74.

In both genders greater percentage of professionals had a work experience of 6 to10 years, while a smaller percentage were in their initial period of service having a work experience of 1 to 5 years. Stress was accentuated in 55.1% of the participants. Presence of diurnal bruxism was reported by 59.2% of the participants. Diurnal bruxism was associated with factors like work experience, work, and stress. But the age factor and gender did not show statistically significant association with diurnal bruxism (Table 2).

Multivariate analyses revealed that individuals with more work experience were less likely to exhibit diurnal bruxism (odds ratio (OR) = 0.04, 95% confidence interval (CI = 0.00–0.43)). Effect of work on the outcome of bruxism was found to be statistically insignificant when age and gender were entered into the logistic regression model. Stress encountered by the professionals showed statistically significant association with diurnal bruxism (*P* < 0.001). The stress was found to be a major risk indicator for diurnal bruxism (OR = 5.9, 95% CI 2.61–13.32) (Table 3). 

## 5. Discussion

This pilot study is to the best of our knowledge, the first of its kind to ascribe the prevalence of diurnal bruxism and elucidate its association with stress- and work-related factors among IT professionals in an industry in India. The total number of participants was 147 (response rate 57%). The hectic working pattern and extended working hours within this IT company gave little room for the authors to collect the sample based on probability and therefore the findings and conclusion should be treated with caution. The study reiterated the vital importance of planning required by researchers when dealing with a group pressed with time. The current study observed that 59.2% of the participants reported to have bruxed their teeth during working hours. Prevalence reported in our study is higher when compared to bruxism reported for other work forces [6, 7] and adult population [8]. Diurnal bruxism was recorded based on self-reports by the participants and was diagnosed based on self-reported awareness of the habit since there are no standardized diagnostic criteria.

The main findings of this study were that more stress and less work experience were significant contributing factors for diurnal bruxism to occur among IT professionals in the present study. Earlier studies on IT professionals in India revealed that 35% of the workforce examined was stressed [15, 16]. Although, this figure is lower than that mentioned in the present study, it unequivocally suggests that stress is a common feature among IT professionals. The odds of experiencing diurnal bruxism were six times more if participants were more stressed compared to those who were less stressed. Ahlberg and colleagues [1] observed that multiprofessional media personnel working with stress were five times more likely to experience bruxism compared to those working without stress. In Brazil, a study conducted among police personnel demonstrated that emotional stress was associated with bruxism regardless of the type of work [6]. The above studies are in conformity with the results of the current study and suggest that stress may cause diurnal bruxism. Stress is encountered due to variety of reasons in one's day-to-day life and the ILO (International Labour Office) in its report has concluded that stress and mental illness are raising problems at workplace [18]. Palmer et al. [19] described that numerous factors like life style, organizational setup, culture, relationships, and role played by the employee contribute to stress levels during work. 

The other risk indicator that showed significance in the logistic regression was number of years of work experience. The likelihood of diurnal bruxism decreased with age. Diurnal bruxism in younger age group could be attributed to stress perceived during extra efforts put in by individuals to establish themselves well in the industry and to emerge as successful professionals. IT professionals in the initial stages of career could perceive more stress and face deficit in coping to stress that are shown to be high among bruxers [9, 20]. Sharma et al. [15] indicated that stress seemed to be accentuated during the initial periods of working with computer among IT professionals. However, the current analyses revealed no such interaction between stress and years of work experience on diurnal bruxism suggesting its independent effect on the dependent variable. We assume that the absence of diurnal bruxism with increasing age among IT professionals may be due to the fact that over the years individuals get acclimatized to their specific type or nature of work and develop coping skills with increase in the work experience. Further investigation is required to explain some of the aforementioned assumptions. 

It is difficult to deduce the cause-effect relationship between stress and diurnal bruxism because of the cross-sectional nature of the present study. Nevertheless, it could be concluded that stress and work experience are plausible risk indicators of diurnal bruxism. The use of questionnaires may underreport the prevalence of diurnal bruxism or other work related factors due to recall bias [21]. Studies based on self-reporting could result in over reporting or underreporting. In this study underreporting is more likely because self-knowledge about bruxism could be less among the participants. The results of this study cannot be extrapolated (external validity) to all IT professionals in Bangalore considering the fact that the sample was derived from only one IT company. This issue requires due attention in further studies.

## 6. Conclusion

The pilot study found that majority of the participating IT professionals reported to have diurnal bruxism. The study also revealed that more stress and a few years of working experience are important risk indicators of diurnal bruxism among IT professionals participating in the present study. The relationship between stress and diurnal bruxism could be described as tentative considering the limitations of the study as discussed. Although the results of this pilot study are imperative, further studies need to consider a prospective design with more representative sample from a wide population of IT professionals and deal with issues relating to validity. IT professionals comprise of an important workforce in today's world and the ramifications of stress in this population may influence their health and also work output. Further research that provides strong evidence can influence the policy issues focusing on the health of these professionals.

##  Conflict of Interests

The authors declare no conflict of interests in the present study.

## Figures and Tables

**Table 1:** Distribution of participants according to age, sex, work experience, work, stress, and bruxism (*N* = 147).

Variables	No. (%)
Age	
20–29 yrs	68 (46.3)
30–39 yrs	61 (41.5)
40 yrs and more	18 (12.2)
Gender	
Males	72 (48.9)
Females	75 (51.1)
Work experience	
1–5 yrs	25 (17.0)
6–10 yrs	79 (53.7)
11 yrs and more	43 (29.3)
Work	
Less	73 (49.7)
More	74 (50.3)
Stress	
Less	66 (44.9)
More	81 (55.1)
Bruxism	
Absent	60 (40.8)
Present	87 (59.2)

**Table 2:** Bivariate analysis showing the association of age, gender, work experience, work, and stress with diurnal bruxism.

Variables	Pearson's chi-square value	*P* value
Age	1.242	0.278
Gender	0.642	0.502
Work experience	11.692	0.003*
Work	5.203	0.029*
Stress	16.559	<0.001*

*Statistically significant, *P* < 0.05.

**Table 3:** Diurnal Bruxism and independent factors among IT professionals (*N* = 147). Multiple logistic regression analyses giving odds ratio and 95% confidence interval.

Factors	*P* value	Odds ratio	95% CI for EXP (B)	
			Lower	Upper
≥33 years^a^	0.596	0.744	0.249	2.221
Males^b^	0.563	1.269	0.566	2.848
Work experience—6 to 10 years^c^	0.526	1.629	0.360	7.368
Work experience— ≥11 years^c^	0.007*	0.042	0.004	0.427
More work^d^	0.151	0.560	0.253	1.237
More stress^e^	<0.001*	5.902	2.614	13.325

*Statistically significant, *P* < 0.05; superscripts under “Factors” represent the reference group for each independent variable: 20–32 years^a^; females^b^; work experience—1–5 years^c^, less work^d^; less stress^e^.

## References

[1] Ahlberg J, Rantala M, Savolainen A (2002). Reported bruxism and stress experience. *Community Dentistry and Oral Epidemiology*.

[2] de Leeuw R (2008). *Orofacial Pain: Guidelines for Assessment, Diagnosis and Management*.

[3] American Academy of Sleep Medicine (2005). *International Classification of Sleep Disorders*.

[4] Lavigne GJ, Huynh N, Kato T (2007). Genesis of sleep bruxism: motor and autonomic-cardiac interactions. *Archives of Oral Biology*.

[5] Seligman DA, Pullinger AG, Solberg WK (1988). The prevalence of dental attrition and its association with factors of age, gender, occlusion, and TMJ symptomatology. *Journal of Dental Research*.

[6] Carvalho ALDA, Cury AADB, Garcia RCMR (2008). Prevalence of bruxism and emotional stress and the association between them in Brazilian police officers. *Brazilian Oral Research*.

[7] Ahlberg K, Jahkola A, Savolainen A (2008). Associations of reported bruxism with insomnia and insufficient sleep symptoms among media personnel with or without irregular shift work. *Head and Face Medicine*.

[8] Kuttila SJ, Kuttila MH, Niemi PM, Le Bell YB, Alanen PJ, Suonpää JT (2001). Secondary otalgia in an adult population. *Archives of Otolaryngology—Head and Neck Surgery*.

[9] Manfredini D, Landi N, Romagnoli M, Bosco M (2004). Psychic and occlusal factors in bruxers. *Australian Dental Journal*.

[10] Van Selms MKA, Lobbezoo F, Wicks DJ, Hamburger HL, Naeije M (2004). Craniomandibular pain, oral parafunctions, and psychological stress in a longitudinal case study. *Journal of Oral Rehabilitation*.

[11] Glaros AG (1981). Incidence of diurnal and nocturnal bruxism. *The Journal of Prosthetic Dentistry*.

[12] Lavigne GJ, Khoury S, Abe S, Yamaguchi T, Raphael K (2008). Bruxism physiology and pathology: an overview for clinicians. *Journal of Oral Rehabilitation*.

[13] Ellis R, Lowell BL (1999). Foreign-origin persons in the U.S. informational technology workforce. *Report III of IT Workforce Data Project*.

[14] e-skills UK, Around the UK—London. http://www.e-skills.com/Around-the-UK/Regions/2088.

[15] Sharma AK, Khera S, Khandekar J (2006). Computer related health problems among information technology professionals in Delhi. *Indian Journal of Community Medicine*.

[16] Suparna K, Sharma AK, Khandekar J (2005). Occupational health problems and role of ergonomics in information technology professionals in national capital region. *Indian Journal of Occupational and Environmental Medicine*.

[17] Casanova-Rosado JF, Medina-Solis CE, Vallejos-Sánchez AA, Casanova-Rosado AJ, Maupomé G, Avila-Burgos L (2005). Lifestyle and psychosocial factors associated with tooth loss in Mexican adolescents and young adults. *Journal of Contemporary Dental Practice*.

[18] Gabriel P, Liimatainen MR (2000). *Mental Health in the Workplace: Introduction*.

[19] Palmer S, Cooper C, Thomas K (2001). Model of organisational stress for use within an occupational health education/promotion or wellbeing programme—a short communication. *Health Education Journal*.

[20] Giraki M, Schneider C, Schäfer R (2010). Correlation between stress, stress-coping and current sleep bruxism. *Head and Face Medicine*.

[21] Kroeger A (1983). Health interview surveys in developing countries: a review of the methods and results. *International Journal of Epidemiology*.

